# Minimally Invasive Lateral Thoracic and Lumbar Interbody Fusion with Expandable Interbody Spacers for Spine Trauma—Indications, Complications and Outcomes

**DOI:** 10.3390/jcm14134557

**Published:** 2025-06-27

**Authors:** Linda Bättig, Gregor Fischer, Benjamin Martens, Anand Veeravagu, Lorenzo Bertulli, Martin N. Stienen

**Affiliations:** 1 Spine Center of Eastern Switzerland, Cantonal Hospital of St. Gallen & Medical School of St. Gallen, 9000 St. Gallen, Switzerland; 2 Department of Neurosurgery, Cantonal Hospital of St. Gallen & Medical School of St. Gallen, 9000 St. Gallen, Switzerland; 3 Department of Orthopedic Surgery, Cantonal Hospital of St. Gallen & Medical School of St. Gallen, 9000 St. Gallen, Switzerland; 4 Department of Neurosurgery, Stanford University, Stanford, CA 94305, USA

**Keywords:** lateral lumbar interbody fusion, lateral thoracic interbody fusion, expandable spacer, ELSA, complications, sagittal parameters, outcome, trauma

## Abstract

**Background**: Lateral lumbar or thoracic interbody fusion (LLIF) is increasingly considered for anterior column reconstruction and restoring segmental lordosis in degenerative, infectious, or deformity conditions. Reports about using LLIF with expandable interbody spacers for spine trauma are scarce. **Methods**: In this retrospective, single-center observational cohort study, we reviewed all patients treated by an expandable LLIF interbody spacer (ELSA^®^ Expandable Integrated LLIF Spacer, Globus Medical Inc) for trauma indication at our spine center between September 2018 and January 2024. The primary outcome measures were fusion rate at 12 months, change in segmental sagittal Cobb angle, and clinical outcome according to the MacNab criteria. Secondary outcomes included adverse events and complications. **Results**: We identified *n* = 21 patients with a mean age of 48.3 (standard deviation (SD) 15.7), 47.6% were female. LLIF was mostly performed at T11/12 (*n* = 4; 19.1%) and T12/L1 (*n* = 10; 47.5%). Indications were AO Spine type A2 (*n* = 4, 19.1%), A3 (*n* = 14; 66.7%) or A4 fractures (*n* = 3; 14.3%) with ligamentous (B2-type) in eight (38.1%) and hyperextension (B3-type) injury in one patient (4.8%). Surgery included the release of the anterior longitudinal ligament in four cases (19.1%). Intraoperative AEs were noted in *n* = 1 (4.8%), postoperative AEs in *n* = 3 (14.3%) at discharge, *n* = 4 (19.1%) at three, and *n* = 2 (9.5%) at twelve months. Segmental sagittal Cobb angle changed from 1.3° (preoperative) to 13.3° at twelve months (*p* < 0.001). Functional outcome was excellent/good in *n* = 15 (71.4%; four missing) at 12 months. The fusion rate at the LLIF level was 100% at the 12-month follow-up. **Conclusions**: LLIF with expandable interbody spacers for spine trauma (off-label use) is safe, promotes solid fusion (100% fusion rate at 12 months), and enables correction of sagittal segmental Cobb angle (mean improvement of 12°), with good or excellent clinical outcomes in most patients (71.4%).

## 1. Introduction

Lateral lumbar or thoracic interbody fusion (LLIF) has been well studied in degenerative and deformity patients, leading to favorable clinical outcomes and excellent correction potential [1,2,3,4,5]. Even though there are fewer studies for this indication, LLIF with short-segment posterior fusion has recently been proposed as a promising alternative to both short and long-segment posterior fusion for traumatic thoracolumbar (TL) injuries [5]. Modifications and extensions of “Trauma LLIF” without interbody spacers or with (hemi-)corpectomies have been proposed in the literature [6,7,8]. Several advantages of LLIF have been stated compared to “posterior only” approaches, including less estimated blood loss (EBL), less damage to paraspinal soft tissue, better alignment, and a higher fusion rate despite a considerably shorter fusion construct in the typically young trauma population [5,7,9].

We recently reported our institutional experience with “Trauma LLIF” as short-segment (monosegmental) anterior–posterior fusion procedure for kyphosing TL-fractures and injuries [5]. We observed the most reproducible and long-lasting reduction in post-traumatic kyphosis, paired with a high rate of fusion and a favorable outcome with a temporary bisegmental, two-staged procedure (posterior instrumentation/fusion with delayed LLIF and hardware removal to release the non-injured caudal motion segment after 3–6 months). In the previous work, we applied solid interbody spacers to promote fusion in most cases (88.6%). Whether or not expandable LLIF spacers may be helpful for particular injury types or patient-specific conditions is currently unclear.

Additionally, we have reviewed and reported our experience with expandable LLIF cages for various indications and have demonstrated its safety [10]. The current work intends to fill the gap of knowledge regarding the indication of expandable interbody spacers in the trauma setting. To our knowledge, no studies report the use of expandable LLIF spacers in the trauma setting, as their use is off-label for this kind of indication. Hence, the current work critically reviews our series of patients in which an expandable LLIF spacer was applied for spine trauma, e.g., to compensate for kyphosing vertebral body (VB) fractures or to close gapping defects in hyperextension injuries [10]. The primary outcome of this study was to evaluate clinical and radiographic outcomes (correction of the Cobb angle) after using expandable interbody spacers in the trauma setting.

## 2. Materials and Methods

### 2.1. Hospital Setting

The Cantonal Hospital of St. Gallen is an academic teaching hospital in Eastern Switzerland, associated with the Medical School of St. Gallen. Approximately 1,000,000 inhabitants live in its catchment area. The Spine Center of Eastern Switzerland is certified (AO Spine Center, Eurospine Surgical Spine Center of Excellence) and is formed of 12 board-certified Neurosurgeons and Orthopedic spine surgeons. We perform about 1000–1100 surgical procedures under general anesthesia annually, and the use and types of implants are unified. LLIF with static spacers was introduced at our center in November of 2011 and is performed about 50–60 times per year on average, primarily for degenerative and deformity indications. The ELSA^®^ Expandable Integrated LLIF Spacer, Globus Medical Inc., Audubon, PA, USA, as an expandable option for LLIF, was introduced to our center in September 2018. The LLIF procedures in this series were performed by five senior spine surgeons or by fellows, senior residents and junior spine surgeons under supervision.

### 2.2. Indication for Surgery and Patient Identification

Fractures of the TL-spine were classified according to the AO Spine, ranging from compression (A-type) over flexion/extension (B-type) to translational (C-type) injuries [11]. Surgery was considered if neurological deficits due to spinal canal stenosis or foraminal compression were present, if the fracture was considered unstable with severe (>25°) or progressive kyphosis or loss of vertebral height, or if patients could not be mobilized due to mechanical back pain, despite adequate analgesic therapy [6]. We consider short-segment posterior instrumentation/fusion with Trauma LLIF as anterior column support in patients with A3-fractures and non-ankylosed spine to avoid the morbidity associated with multilevel posterior spinal fusion [12]; short-segment posterior instrumentation/fusion with corpectomy was generally performed in patients with A4-fractures [10]. Some selected patients with superior burst-split fractures (A3.2.1 according to Magerl [13]) were also considered for LLIF in case of low fracture comminution. A single simple split fracture line down to the lower disc level is seen in these fractures, which are classified as A4 according to the AO Spine. However, no disc material has ruptured into the vertebral body preventing healing of the two vertebral body parts. Long-segment posterior instrumentation/fusion with Trauma LLIF was performed in patients with hyperextension injuries (B3-type), ankylosed spines and large traumatic gaps in the anterior column.

Unique patient numbers (UPNs) of patients where an ELSA^®^ spacer was employed from November 2011 until January 2024 were identified by electronic review of our hospital’s purchasing department. We then cross-checked the surgical program for any Trauma LLIF procedure to ensure no patient was missed. Ventro-dorsal, dorso-ventral, and dorso-ventro-dorsal approaches for fusion were included if an expandable LLIF spacer for a traumatic spine injury had been used.

### 2.3. Data Collection and Variables

Three surgeons (LB, GF, MNS) retrospectively reviewed the electronic patient charts to extract relevant information. Baseline information included sex, age, BMI, smoking status, ASA grade, Charlson comorbidity index [14], and the Canadian clinical frailty scale (ranging from 1 (very fit) to 9 (terminally ill)) [15], as well as the AO Spine classification for TL-trauma [16].

Baseline sagittal segmental lordosis (SL)/Cobb angle (e.g., upper endplate of T11 vertebra to lower endplate of T12 vertebra for the T11/12 segment), as well as spino-pelvic parameters including pelvic incidence (PI), lumbar lordosis (LL; from L1-S1), pelvic tilt (PT) and C7-sagittal vertebral axis (C7 SVA) were measured, whenever available, using standing scoliosis X-ray images. The Roussouly type of spinal geometry and “ideal LL” were calculated using a web-based app (https://spinebit.io (accessed on 10 February 2024)), which is based on the formulas by Le Huec and the European Spine Study Group [17,18,19].

Surgical parameters included the number of segments fused (e.g., T11-L1), LLIF segment (e.g., T11/12), intraoperative release of the ALL, length of the surgery (in minutes), EBL (in ml; defined by anesthesiologists from suction drainage and bloody swabs) and intraoperative AEs.

Postoperative AEs at the time of discharge from hospital were considered, as documented by the discharge report, classified according to the Therapy–Disability–Neurology (TDN) scoring system, which considers not only the therapeutical consequence of an AE, but also the impact on patient’s well-being/severity [20]. The same was extracted from the outpatient clinic reports at 3 months (3 M; AEs occurring between discharge and 3 M follow-up) and at 12 months (12 M; AEs occurring between 3 M and 12 M follow-up). Clinical outcome was graded according to MacNab into four categories, as best estimated from the outpatient clinic report letter (excellent, good, fair, poor) and available Patient-Reported Outcome Measures (PROMs) [21,22,23].

Postoperative radiological outcomes at 3 M and 12 M again included SL, LL, PI, PT, and C7 SVA. Pseudarthrosis at the LLIF level was defined as a failed attempt at spinal fusion, manifesting with a new onset of axial or radicular pain weeks to months after the index operation [24]. Diagnosis of pseudarthrosis was based on both the clinical presentation and available imaging studies (X-ray, computed tomography (CT) and Single Photon Emission Computed Tomography (SPECT)) after ruling out other causes of persistent pain.

### 2.4. Surgical Technique and Special Considerations

After positioning the patient strictly lateral under fluoroscopic control, we typically approached the disc space from the left side. In the lower lumbar spine (L2/3–L3/4), an anterior-to-psoas or a trans-psoas approach utilizing directional electromyography (EMG) was used. The junctional levels Th12/L1 and L1/2 may require a phrenicotomy. For the thoracic segments (Th11/12 and higher), either a retro-pleural access or a mini-thoracotomy was used. After fluoroscopic confirmation of the correct location (and serial dilatation in the lumbar spine), the table-mounted MaXcess^®^ retractor (NuVasive, Inc., San Diego, CA, USA) was brought in and fixed firmly. The disc was resected with a combination of curettes and rongeurs; the Cobb elevator was used to release the contralateral annulus. If needed, the ALL was cut with a knife or a chisel after applying soft tension with an expandable sizer, thus optimizing controlled ALL release and protecting the blood vessels anterior of the spine. If posterior fragments compromising the spinal canal were present and could not be resected in the posterior procedure, they were removed during the lateral approach after being mobilized ventrally. After trialing with a sizer, the definitive expandable spacer was brought in and opened under fluoroscopic control until coming into firm contact with the endplates. The spacer was fixed to one or both vertebral bodies using an integrated screw to prevent it from migrating anteriorly, especially when hyperlordotic spacers were used and/or the ALL had been released. The spacer was further expanded until the desired height and lordosis were achieved. The graft chamber of the spacer was filled with biologics and/or bone chips using a funnel.

**Figure 1:**
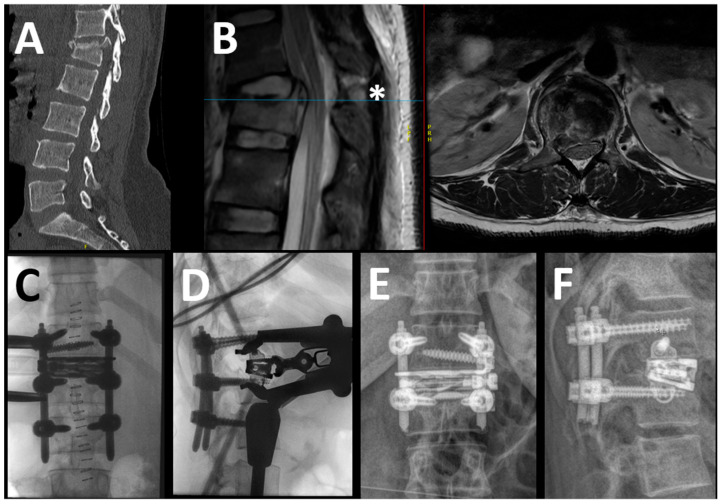
In this example, the case of a 39-year-old woman with a B2 injury at T12-L1 level (L1: A4) is reported. The patient presented without neurological deficits. In the upper row, the preoperative sagittal view of the CT scan (**A**) and the sagittal and axial T2 MRI (separated by the red line) (**B**) are shown, demonstrating the complete burst fracture of L1 along with the lesion of the posterior tension band, the blue line indicating the height of the axial imaging (*); in this case, there was no significant compression of the conus despite the slightly displaced posterior wall fragment. The intraoperative (**C**,**D**) and 1-year postoperative (**E**,**F**) images are shown in the lower row. In this case, we placed monoaxial screws percutaneously (no need for canal decompression) to effectively restore the alignment and performed the LLIF in the same anesthesia (=one-staged, temporary bisegmental). Notice the position of the screws in L1, which act as additional support for the expandable spacer.

We do not perform stand-alone LLIF in trauma patients. Hence, all patients underwent additional posterior instrumentation/fusion, usually before the LLIF procedure. Other than for degenerative/deformity cases where the lateral approach is often chosen first, in trauma, we prefer to start with the posterior part, either open or percutaneously, to stabilize the spine, decompress, and clear the spinal canal from bone fragments if needed, and reduce the kyphosis. Moreover, we try and intentionally place the screws of the fractured vertebra close to the fractured (upper) endplate, so that the expandable spacer can be opened until resting on the pedicle screws should the endplate destruction be advanced (see Figure 1). Monoaxial screws (for percutaneous approach) or polyaxial screws with a monoaxial feature (for open approach) are used to allow for angle-stable distraction without increasing segmental kyphosis.

### 2.5. Statistical Analysis

We used Stata (StataCorp LLC, College Station, TX, USA) v14.2 for Mac and employed mostly descriptive statistics, reporting results as mean (standard deviation) or count (per cent). For the analysis of sagittal spinal parameters over time, outcomes were compared to the preoperative value using paired t-tests to analyze sagittal spinal parameters over time. Probability values < 0.05 were considered statistically significant.

### 2.6. Ethical Considerations

The institutional review board of Eastern Switzerland approved the study (BASEC ID 2023-01343). Retrospective collection, analysis, and publication of anonymized patient data were allowed with an institutional waiver for informed consent.

## 3. Results

### 3.1. Patient Cohort

We identified 503 patients in whom we performed LLIF at 732 levels. In 21 patients (4.2%), an expandable LLIF spacer was used for a traumatic indication in the TL-junction or the lumbar spine, which built the final sample for this analysis. Of the 87 patients treated with Trauma LLIF in the studied time interval, 21 benefitted from the use of an expandable spacer (24.1%). All patients received LLIF for one single motion segment (21 levels treated).

The mean age of the cohort was 48.3 years (standard deviation (SD) 15.7), 47.6% were female and 28.6% active smokers. The cohort had a low surgical risk overall (66.7% ASA grade I or II), few comorbidities (85.7% CCI 0 to 1), and low frailty scores (66.7% very fit or well). According to the AO classification, four patients suffered from A2 fractures, 14 from A3 fractures, and three from A4 fractures. In about 40% of patients, additional ligamentous (B2-type; 38.1%) or hyperextension injury (B3-type; 4.8%) was evident. More detailed baseline demographic information can be found in Table 1.

Table 2 summarizes the key surgical parameters. LLIF was performed in the thoracic spine (T11-L1) in 14 (66.6%) and in the lumbar spine (L1-L4) in seven (33.3%) patients. Most were mono- or bi-segmental fusion procedures (*n* = 15; 71.4%) employing either lordotic (67.7%) or hyperlordotic spacers (9.5%) without deliberate ALL release (*n* = 17; 80.9%). The mean operative time was 233 (SD 88) min, and EBL was 313 (SD 231) ml.

### 3.2. Follow-Up and Reasons for Missing Data

The mean length of hospital stay was 9.1 (SD 5.18) days. The 3 M follow-up was completed by 19 (90.5%) patients at a mean of 91 days postoperative. The 12 M follow-up was completed by 17 (81%) patients at a mean of 311 days postoperative. Reasons for drop-out during follow-up included surgery performed within <1 year, patients moving away from the area (e.g., patients living abroad with trauma during a vacation in the region) or seeking care at another facility.

### 3.3. Radiological Outcomes

Table 3 illustrates the evolution of the sagittal radiological parameters over time. There was a significant postoperative increase in LL and SL, which was preserved over time. Moreover, PT and C7 SVA declined in the postoperative period.

With ALL release, the change in pre- to immediate postoperative SL was greater (−2.8 ± 13.6° → 16.4 ± 13.5°, *p* < 0.001) than without ALL release (4.3 ± 17.1° → 10.5 ± 12.3°, *p* = 0.006).

### 3.4. Clinical Outcomes

The rates of excellent or good outcomes at 3 M and 12 M were (81% (two patients missing) and 71.4% (four patients missing), respectively (Table 4). Postoperative AEs were observed in *n* = 3 (14.3%) at discharge, *n* = 4 (19.1%) at 3 M and *n* = 2 (9.5%) at 12 M; detailed information on the AE type and severity is displayed in Table 4. We observed no pseudarthrosis at the LLIF level during follow-up (Table 4).

## 4. Discussion

In this retrospective observational cohort study, we focused on patients undergoing LLIF with an expandable interbody spacer in the setting of spine trauma. We found that both SL and total LL could be effectively restored postoperatively and remained stable over the follow-up period. The “Trauma LLIF” was typically used in a mono- or bisegmental fashion, striving for short fusion constructs to avoid complications and morbidity associated with long fusion constructs, e.g., early development of adjacent segment disease (ASD), proximal junctional failure (PJF) and functional restrictions. The intra- and postoperative AE rates in this series ranged between 10 and 20% and were mainly not directly associated with the surgery (i.e., macrohematuria). Most patients reported an excellent or good outcome at 3 and 12 months postoperatively, and we did not detect any patient with pseudoarthrosis at the LLIF level. Altogether, “Trauma LLIF” with an expandable interbody spacer and posterior pedicle-screw-based fusion appeared to be a safe and effective surgical treatment option.

### 4.1. Why “Trauma LLIF”?

General goals of surgery for spine trauma include decompression of injured neural elements, fixation, or fusion of an unstable fracture, and reduction or correction of post-traumatic deformity [25]. The ideal approach (anterior, posterior, lateral, or combined) for this is still controversial, but different techniques are likely beneficial in particular situations.

The use of LLIF for spine trauma is rarely described in the literature. We recently described our experience with “Trauma LLIF” using non-expandable static spacers [5]. We found that Trauma LLIF was a safe procedure, which led to a long-lasting reduction in segmental kyphosis by short-segment, mono-segmental fusion. However, interbody fusion by a posterior approach or (hemi-)corpectomies is often described for TL injuries [6]. In a comparative small case series of osteoporotic and traumatic injuries of the lumbar spine (L2-L5), Sasaki et al. found that LLIF (*n* = 3) procedures with percutaneous pedicle screw fixation a few days later resulted in less blood loss (133 vs. 593 mL) and shorter surgical time (243 vs. 396 min) compared to open posterior lumbar interbody fusion (PLIF; *n* = 3) [7]. No pseudoarthrosis was detected in their case series, and patients in both treatment groups showed significant improvement in low back pain, reported at a 3-month follow-up. However, the treatment applied in both the LLIF and the PLIF group did not correct the deformity, and the authors described the loss of segmental correction in all PLIF patients and—to a lesser extent—also in the LLIF patients [7]. As opposed to these authors, we advocate a treatment that stabilizes the spine and corrects the deformity first, using pedicle screws with a monoaxial feature and segmental distraction, followed by LLIF either immediately (single-staged, temporary bisegmental) or after approximately 3–5 months, combined with the release of the lower motion segment in the same anesthesia (two-staged, temporary bisegmental) [5]. The more favorable results observed in our cohort are likely due to these differences in the treatment algorithm. However, the younger age in the present patient cohort with presumably better bone quality may have also had a positive effect. Failure to heal has been described to follow one of four main reasons: (1) the characteristics of the vertebral bone (including characteristics of the individual, e.g., age, bone quality, etc.), (2) the properties of the interbody device (e.g., material and type), (3) the interaction between cage and further fixation devices (plate or screws), and (4) the surgical technique.

The LLIF approach here offers certain advantages compared to other approaches. Compared to PLIF and TLIF, LLIF offers a shorter operative time and less EBL to remove the disc and place the spacer while keeping intact both the anterior and posterior longitudinal ligaments besides the posterior soft tissues [7]. Due to the possibility of inserting wide foodprint spacers, there is a lower risk of spacer subsidence [9]. Compared to ALIF approaches, it is associated with less postoperative pain, less estimated blood loss, and less morbidity (vascular injuries, retrograde ejaculation) [9,26]. Additionally, it has a strong potential to realign the anterior column and correct sagittal imbalance [10]. These are all features that make it an ideal solution for a damaged, unstable, and deformed spine, as is often encountered after significant trauma.

### 4.2. Which Type of Spine Trauma Is Suitable for “Trauma LLIF”?

**Figure 2:**
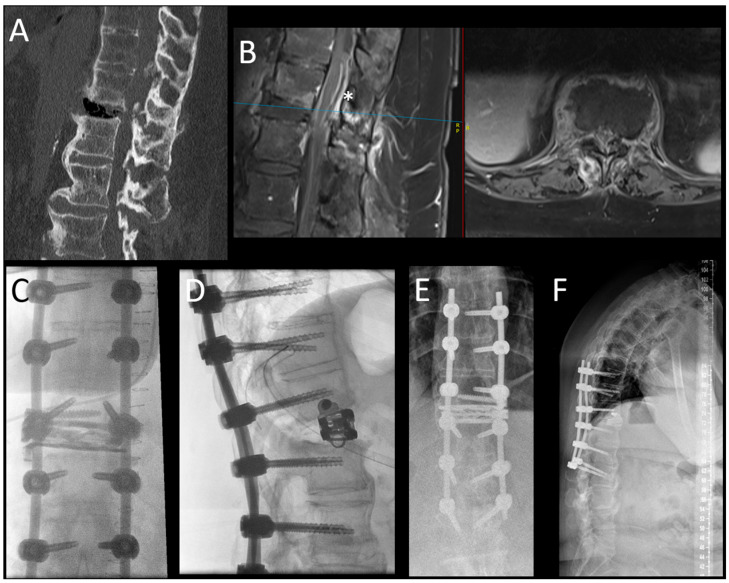
This picture demonstrates the case of a 59-year-old woman who presented with a B3-type injury of the T12-L1 segment in the setting of an ankylosed spine (Bechterew’s disease). In the upper row, the sagittal view of the CT scan (**A**) demonstrates the extension injury of the segment with massive air collection in the ruptured disc space and the typical findings of a Bechterew’s disease with ossification of ALL and the posterior ligaments. In the sagittal and axial MRI scan (separated by a red line) (**B**), an epidural hematoma with compression of the conus (*) can be appreciated. The blue line indicates the height of the axial imaging on the sagittal imaging. The intraoperative (**C**,**D**) and postoperative (**E**,**F**) images are shown in the lower row. Here, the posterior stabilization was performed in an open fashion because a spinal canal decompression was needed; the construct was chosen as long-construct fusion (3 levels above and below the injury) due to the ankylosed spine. In this case, the LLIF procedure was performed in a separate anesthesia. With the ELSA expandable spacer, the gapping defect at the level of the disc T12-L1 was filled, and the spacer was secured with the additional screw to avoid dislocation. The additional anterior column support helped to keep the lordosis of this segment, gained by the injury, in a globally kyphotic spine.

**Figure 3:**
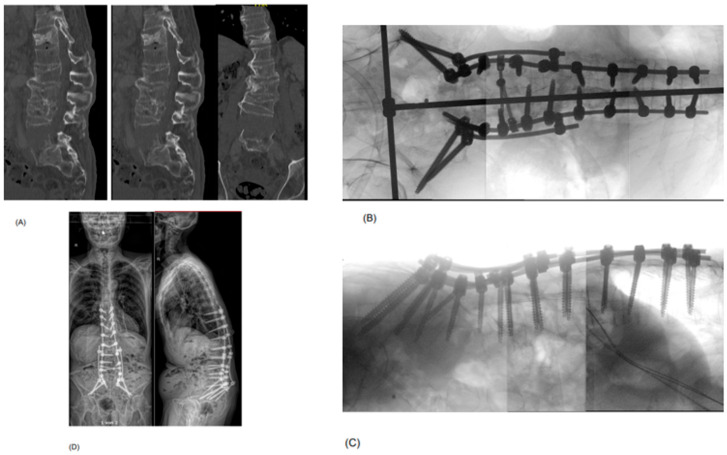
Example of a 73-year-old patient who presented with a B3-type hyperextension injury at the level of the L5 vertebral body in an ankylosed spine, with additional traumatic injuries to the lumbar spine (old L4 incomplete burst fracture (A3) with acutely ruptured L3/4 disc and L4 pedicle fracture, acute Th12 complete burst fracture (A4)) and abdominal organs (e.g., suspected common iliac vein injury) after a motorcycle accident. He was neurologically intact. As the injury was at the level of the L5 vertebral body, an LLIF to fill up the gapping hyperextension injury was not considered possible. Additionally, the retroperitoneal hemorrhage by a venous injury—treated conservatively—hindered an anterior or lateral approach to provide anterior column support by a spacer. (**A**) shows the preoperative sagittal and axial CT scan post-trauma, demonstrating the extension injury of L5 (chance fracture with craniocaudal dislocation), complete burst fracture of Th12, rupture of the disc space L3/4 and fracture of the pedicle L4 on the right side. (**B**,**C**) demonstrate the intraoperative imaging after stabilizing the fractures by a T10-pelvis fusion construct with a four-rod system (S2Ai and iliac screws). (**D**) shows the standing whole-spine EOS X-ray in ap and lateral view at 3 months postoperative. Note how the gapping defect of L5 has closed to some extent over time, without the anterior column support, resulting in loss of lordosis (38.5°) compared to the intraoperative view.

Our patient cohort can be divided into two subgroups based on the trauma mechanisms and the AO Spine TL-injury classification [27]. The first group contains the kyphosing compression (A1) and burst (primarily A3, few A4) fractures with or without flexion-distraction injury (B1 and B2; Figure 1). The other group contains extension injuries (B3), which are primarily present in patients with ankylosing comorbidities, such as diffuse idiopathic skeletal hyperostosis (DISH) or Bechterew’s disease (Figure 2). While in the first group, the expandable spacer is used to restore the loss of lordosis to the desired degree (by selecting from parallel (0°) to hyperlordotic (15–30°)), it is used to fill significant wedge-shaped fracture gaps in the second group. Albeit less frequent than the first group, it may happen during the treatment of B3 fractures that the posterior pedicle screw fixation in prone position does not allow for reducing the fracture gap by actively kyphosing the spine. In some patients with pre-injury kyphosis, the gain in lordosis by the traumatic “osteotomy” allows for a much more upright posture, hence keeping this position is desired, but the substantial defect in the anterior column may predispose for segmental mobility, non-union and screw loosening. The expandable spacer can help reconstruct the anterior spinal column, realign it, and help with solid anterior–posterior fusion despite a reasonably limited fusion construct. In patients with ankylosed spines and hyperextension trauma where a large defect of the anterior column cannot be filled up by an interbody spacer, extensive multiple-rod constructs may be required (Figure 3). Examples of both Trauma LLIF patient types are provided in Figure 1 and Figure 2.

### 4.3. Why and When Should Expandable Interbody Spacers Be Used for “Trauma LLIF”?

Our rationale is to treat traumatic TL fractures with a short construct. We intend to achieve a monosegmental fusion, whenever possible, to prevent the morbidity associated with longer fusion constructs in young patients, including but not limited to functional disability and stiffness and premature development of ASD or PJF [12,28]. When staying short with the fusion construct in an unstable injury setting, it is beneficial to add anterior support, which helps to prevent hardware failure due to the high biomechanical forces affecting the screws [12,28].

Static interbody spacers function well as a standard solution for LLIF in the trauma setting. However, expandable spacers offer some distinct advantages, which we briefly highlight in the following. Those spacers are available in hyperlordotic versions of 5–20° or even up to 15–30°, which can be required when trying to compensate for the kyphosing effect of a burst fracture or when filling up an extension fracture gap [10]. In contrast, static spacers are only available up to 15°. Compared to static spacers, where spacer height is often limited to a maximum of 12 mm (as they have been developed for degenerated, typically collapsed disc spaces), expandable spacers can be opened several millimeters more, achieving spacer heights of 18 or even 20 mm. This can be decisive when filling out the typically significant anterior column defects—in young patients without pre-injury disc degeneration or in elderly patients with gapping extension fractures—as the static spacers with limited height may not sufficiently fill them out. A further advantage is that it can be inserted in the disc space in a closed fashion and then be carefully expanded in situ, preventing further injury to the already traumatized spine and from mobilizing possible fragments into the spinal canal [10].

In contrast, static spacers must be inserted with greater force to achieve a press fit. The fractured endplate is better protected by the expandable spacer’s mode of insertion, which may lead to lesser spacer subsidence. As subsidence of the spacer into the injured endplate, to a certain degree, cannot be prevented, we plan the screw position accordingly in anticipation of this problem. The monoaxial pedicle screws of the fractured vertebra are positioned close to the upper (injured) endplate, offering additional support for the expandable spacer to rest upon should the endplate give way during spacer expansion. Cement augmentation of the screws offers supplementary support in selected elderly patients with lower bone quality, creating a solid ground for the expandable spacer.

In the literature, expandable interbody spacers are associated with low subsidence rates and a substantial lordosis correction potential, similar to or even higher than solid spacers [6,10]. However, those devices have almost exclusively been studied for degenerative causes, not for the trauma indication. In a retrospective series of *n* = 62 patients with degenerative indications, Li et al. compared expandable to static spacers for degenerative disc disease and found a higher rate of spacer subsidence in the static group (16.1 vs. 6.7%, *p* < 0.05) and better outcomes in visual analogue scale (VAS) of leg and back pain, respectively, as well as Oswestry disability index (ODI) at 6 and 12 months [29]. Frisch al. also found significant differences in both outcome and radiological features in their retrospective cohort study on *n* = 29 patients with degenerative disc disease. The spacer subsidence rate in their series was significantly higher in the static group (16.1% vs. 0%, *p* < 0.01) [30]. The group around Huo et al. published their prospective observational study of 98 patients in 2023, where the spacer subsidence rate was higher at all times of the follow-up in the static groups (12 months: 4% vs. 20% *p* = 0.004), leading to similar 12-month fusion rates (94.0% vs. 82.9%%, *p* = 0.039), however [31]. Studies examining expandable LLIF spacers in the trauma setting are needed to provide evidence that they can be safely used, even though they are often not approved (off-label) for this indication.

The downsides of expandable spacers include their higher costs compared to static spacers, which must be weighed against the potential benefit. No cost–benefit analyses are currently available. Moreover, the unmindful use of expandable spacers may result in overexpansion, which is clinically relevant as it may worsen endplate injury and facilitate consecutive spacer subsidence. If subsidence, which is to some extent expected in the trauma indication with VB fractures, is not anticipated and screw placement is chosen accordingly (see above), it may result in loss of correction, non-union during follow-up and need for surgical revision [10].

### 4.4. Limitations of and Caveats for “Trauma LLIF”

LLIF for the trauma indication has some pitfalls which need to be considered. The presence and position of posterior wall fragments are important when planning the case. If a fragment protrudes into the spinal canal, the position of the reasonably sized LLIF spacer in the anterior third of the disc space should be aimed to avoid its accidental posterior movement. An expandable spacer should also be considered in patients with posterior wall fragments, as the spacer can be introduced more gently without hammering force. Moreover, in multi-organ injuries, adhesions from hematomas, injured soft tissue or vessels can make the LLIF approach more difficult [9], and other surgical techniques should be prioritized. The lumbosacral plexus can be injured, especially in trans-psoas approaches [9], which should ideally be performed using electrophysiological monitoring. For LLIF in the TL-junction, especially for the Th12-L1 and L1-2 segments, knowledge of the diaphragm anatomy, rib spacer, and pleura is required [32]. For trans-thoracic LLIF approaches, a Valsalva maneuver conducted before closing the parietal pleura can help detect possible lung injuries and reduce the risk of a tension pneumothorax [32]. Lastly, Trauma LLIF is best performed in patients with acute or subacute spine trauma, as the motion segment is still mobile in the spine, and a deformity can better be corrected. In patients presenting delayed (after several months) with post-traumatic deformities, an adequate segmental correction may require the deliberate release of the ALL, sometimes even a posterior–anterior–posterior approach, which increases the risk for complications [25]. In AO Spine type A4 fractures, an LLIF with an expandable spacer might not be enough to heal the burst component due to severe fracture comminution and highly unstable endplates. Corporectomy using a posterior a lateral approach via mini-lumbotomy/thoracotomy may be required for those. In A3 fractures requiring less kyphotic correction, LLIF using static interbody spacers or posterior only fusion approaches are likely sufficient [5].

### 4.5. Strengths

We report the use of a novel technology for a highly under-reported “off-label” indication. Today, there is minimal data on the use of LLIF for trauma in general, and even more so with expandable spacers, which this report combines. Further strengths include including relevant clinical and radiological outcome measures over a 12-month follow-up with reasonably few missing data points.

### 4.6. Limitations

The weaknesses of this study are its retrospective character and limited sample size, which could not be avoided as we performed Trauma LLIF with expandable spacers on carefully selected patients. The small sample prevented us from conducting interesting subgroup analyses, e.g., based on the trauma mechanism or the AO classification injury type. Furthermore, selection bias likely applies, as we chose individually in which patient to implant an expandable versus a static spacer. Four patients have not completed the follow-up at 12 months for various reasons; one was seeking care at another facility. This might lead to overestimating good outcomes in the rest of our cohort. PROMs were only implemented in our center in 2022, so those were unavailable for all patients included in this study. Hence, we used the MacNab criteria with a simple four-tier scale to classify outcomes for this study but included the PROM information whenever disposable [10]. The use of the MacNab criteria might have overestimated good outcomes due to the subjectivity of the score.

Additionally, not all patients received a postoperative CT scan to control the extent of fusion, as standing X-ray in two planes are the standard of care at our center for patients doing well. However, in patients with suspicion of hardware problems or pseudarthrosis, as well as before planned (partial) removal of spacers to release a non-injured motion segment during follow-up, CT scans or single photon emission computed tomography (SPECT) scans are requested. Further cohort studies to validate our findings are desirable, as are analyses including a control group to compare, e.g., solid vs. expandable spacers. Lastly, the follow-up period of one year, on average, is not enough to control pseudoarthrosis, subsidence and the impact of osteoporosis on the construct over more extended periods. Future studies should concentrate on long-term follow-ups on this subject.

### 4.7. Implications for Practice

Considering the results of this study, the recent new literature [5,10], and our positive experience using expandable LLIF spacers, we will continue to consider it in specific spinal trauma patients. As more surgeons are becoming familiar with and applying the LLIF technique in their practice, it would be interesting to compare the Trauma LLIF technique with other frequently used approaches in traumatic spine injury, e.g., short- or long-segment posterior-only instrumentation/fusion. Until better evidence regarding the comparative effectiveness is available, we suggest using expandable LLIF spacers in selected patients only: (1) young and active patients who will benefit from ultra-short (monosegmental) fusion or who require a high degree of segmental deformity correction, or (2) patients with extension injuries of an ankylosed spine and significant wedge-shaped defects of the anterior column.

## 5. Conclusions

In this series, LLIF for trauma using an expandable titanium interbody spacer was safe, promoted solid fusion in 100% of the patients, and had a high correction potential for kyphotic deformity, which remained stable over the one-year follow-up period.

## Figures and Tables

**Table 1:** Baseline demographic information. Results are presented as mean (standard deviation, range) or count (per cent). SS = sacral slope, ASA = American Society of Anesthesiologists, AO = Arbeitsgemeinschaft für Osteosynthesefragen.

Baseline Demographic Information	
Age in years	48.3 (15.7, 17–76)
Sex	
Female	10 (47.6%)
Male	11 (52.4%)
ASA risk scale	
I	11 (52.4%)
II	3 (14.3%)
III	7 (33.3%)
IV	- (0%)
Charlson comorbidity index	0.7 (1.6, 0–5)
0–1	18 (85.7%)
2–3	1 (4.8%)
4 or higher	2 (9.5%)
Canadian Clinical Frailty Index	
Very fit or well	14 (66.7%)
Managing well or vulnerable	4 (19.1%)
Mildly or moderately frail	2 (9.5%)
Severely or very severely frail	1 (4.8%)
Smoking status	
Active smoker	6 (28.6%)
Former smoker	2 (9.5%)
Nonsmoker	13 (61.9%)
AO Spine Classification	
A2 fracture	4 (19.1%)
A3 fracture	14 (66.7%)
A4 fracture	3 (14.3%)
Additional B2 injury	8 (38.1%)
Additional B3 injury	1 (4.8%)
Roussouly type of spinal geometry	
Type 1 (SS < 35°)	6 (28.6%)
Type 2 (SS < 35°)	4 (19.1%)
Type 3 (35° < SS < 45°)	4 (19.1%)
Type 4 (SS > 45°)	7 (33.3%)
Total	21 (100%)

**Table 2:** Surgical parameters. Results are presented as mean (standard deviation, range) or count (percent). ALL = anterior longitudinal ligament; LLIF = lateral lumbar or thoracic interbody fusion. * Complications: asymptomatic cement leakage and cerebrospinal fluid leak in one patient.

Surgical Parameters	
LLIF segment	
T11-12	4 (19.1%)
T12-L1	10 (47.5%)
L1-2	2 (9.5%)
L2-3	4 (19.1%)
L3-4	1 (4.8%)
Number of fused segments	2.1 (1.5, 1–6)
Mono-/bisegmental	15 (71.4%)
3–7 segments	6 (28.6%)
ALL release	
Yes	4 (19.1%)
No	17 (80.9%)
Length of surgery, in minutes	233 (87.6, 96–409)
Estimated blood loss, in milliliters	313 (231, 20–1000)
Type of interbody spacer	
Parallel (0° lordosis)	1 (4.8%)
Anatomical (6° lordosis)	4 (19.1%)
Lordotic (5–20° lordosis)	14 (67.7%)
Hyperlordotic (15–30° lordosis)	2 (9.5%)
Intraoperative AEs	
No	20 (95.2%)
Yes, type:	1 (4.8%)
Vascular injury	-
Nerve injury	-
Spacer subsidence	-
Other *	1 (4.8%)
Total	21 patients/21 levels (100%)

**Table 3:** Sagittal radiological parameters over time. Results are presented in degrees (°) or centimeters (cm) as mean (standard deviation, range). The level of significance is indicated by comparing the value at follow-up with the preoperative value. C7 SVA = C7 sagittal vertical axis; LL = lumbar lordosis; PI = pelvic incidence; PT = pelvic tilt.

Parameter	Preoperative	Discharge	3 Months	12 Months
PI, in °	53.0 (12.6)	-	-	-
LL, in °	32.7 (18.5)	45.9 (9.2) *p* < 0.001	45.3 (13.4) *p* < 0.001	43.3 (15.0) *p* < 0.001
PT, in °	23.6 (10.3)	18.8 (7.7) *p* < 0.001	20.7 (9.7) *p* = 0.040	19.6 (9.1) *p* = 0.003
Segmental lordosis, in °	1.3 (16.0)	13.0 (13.1) *p* < 0.001	12.7 (13.8) *p* < 0.001	13.3 (14.5) *p* < 0.001
C7 SVA, in cm	8.0 (6.4)	5.6 (4.3) *p* = 0.001	4.9 (4.2) *p* = 0.005	6.6 (5.1) *p* = 0.114
Total	*n* = 21 patients/*n* = 21 levels (100%)			

**Table 4:** Clinical outcomes over time. Results are presented as count (per cent). * Adverse events (AEs) are indicated as occurring between surgery and discharge, discharge, and 3 months, or between the 3- and 12-month follow-up. ** at the LLIF level. TDN = Therapy–Disability–Neurology grading scale of complications.

Parameter	Discharge	3 Months	12 Months
Functional outcome	-		
Excellent		11 (52.4%)	8 (38.1%)
Good		6 (28.6%)	7 (33.3%)
Fair		1 (4.8%)	1 (4.8%)
Poor		1 (4.8%)	1 (4.8%)
Missing data		2 (9.5%)	4 (19.1%)
Postoperative AE *			
No	18 (85.7%)	15 (71.4%)	15 (71.4%)
Yes	3 (14.3%)	4 (19.1%)	2 (9.5%)
Missing data	- (0%)	2 (9.5%)	4 (19.1%)
TDN grading scale			
Grade 1	- (0%)	1 (4.8%)	- (0%)
Grade 2	2 (9.6%)	1 (4.8%)	- (0%)
Grade 3	1 (4.8%)	2 (9.5%)	1 (4.8%)
Grade 4	- (0%)	- (0%)	- (0%)
Grade 5	- (0%)	- (0%)	1 (4.8%)
Missing data	- (0%)	2 (9.5%)	10 (15.8%)
Pseudarthrosis **	-		
No		21 (100%)	21 (100%)
Yes		- (0%)	- (0%)
Total	*n* = 21 (100%)		

* Type of AEs: at discharge: anemia, pneumonia, and macrohematuria, pneumothorax; at 3 months: iliosacral joint pain, psoas weakness due to lateral approach, infection, posterior wound dehiscence; at 12 months: bursitis trochanterica.

## Data Availability

The authors will make the raw data supporting this article’s conclusion available upon request.

## Abbreviations

The following abbreviations are used in this manuscript:

AE	Adverse event
ALIF	Anterior interbody fusion
AO	Arbeitsgemeinschaft für Osteosynthesefragen
ALL	Anterior longitudinal ligament
ASA	American Society of Anesthesiologists
ASD	Adjacent segment degeneration
C7 SVA	C7- sagittal vertebral axis
CCI	Charlson Comorbidity Index
CT	Computed tomography
DISH	Diffuse idiopathic skeletal hyperostosis
EBL	Estimated blood loss
EMG	Electromyography
IRB	Institutional review board
LL	Lumbar lordosis
LLIF	Lateral lumbar (or thoracic) interbody fusion
LOS	Lengths of hospital stay
ODI	Oswestry Disability Score
PI	Pelvic incidence
PJF	Proximal junctional failure
PLIF	Posterior interbody fusion
PROM	Patient reported outcome measure
PT	Pelvic tilt
SD	Standard deviation
SL	Segmental lordosis
SPECT	Single photon emission computed tomography
SS	Sacral slope
TDN	Therapy–Disability–Neurology
TL	Thoracolumbar
TLIF	Transforaminal interbody fusion
UPN	Unique patient number
VAS	Visual Analogue Scale
VB	Vertebral body
